# Gender differences in self reported long term outcomes following moderate to severe traumatic brain injury

**DOI:** 10.1186/1471-2377-10-102

**Published:** 2010-10-28

**Authors:** Angela Colantonio, Jocelyn E Harris, Graham Ratcliff, Susan Chase, Kristina Ellis

**Affiliations:** 1 Toronto Rehabilitation Institute, 550 University Avenue, Toronto, Canada; 2 Department of Occupational Science and Occupational Therapy, University of Toronto, 500 University Avenue, Toronto, Canada; 3 HealthSouth, Harmarville Rehabilitation Center, 320 Guys Road, PA, US; 4 Working Order, 1650 Main Street, Sharpsburg, PA, US

## Abstract

**Background:**

The majority of research on health outcomes after a traumatic brain injury is focused on male participants. Information examining gender differences in health outcomes post traumatic brain injury is limited. The purpose of this study was to investigate gender differences in symptoms reported after a traumatic brain injury and to examine the degree to which these symptoms are problematic in daily functioning.

**Methods:**

This is a secondary data analysis of a retrospective cohort study of 306 individuals who sustained a moderate to severe traumatic brain injury 8 to 24 years ago. Data were collected using the Problem Checklist (PCL) from the Head Injury Family Interview (HIFI). Using Bonferroni correction, group differences between women and men were explored using Chi-square and Wilcoxon analysis.

**Results:**

Chi-square analysis by gender revealed that significantly more men reported difficulty setting realistic goals and restlessness whereas significantly more women reported headaches, dizziness and loss of confidence. Wilcoxon analysis by gender revealed that men reported sensitivity to noise and sleep disturbances as significantly more problematic than women, whereas for women, lack of initiative and needing supervision were significantly more problematic in daily functioning.

**Conclusion:**

This study provides insight into gender differences on outcomes after traumatic brain injury. There are significant differences between problems reported by men compared to women. This insight may facilitate health service planners and clinicians when developing programs for individuals with brain injury.

## Background

According to the Center for Disease Control and Prevention, 1.7 million Americans sustain a traumatic brain injury (TBI) each year [1]. Advances in medical technology and care have led to increasing numbers of individuals surviving TBI and requiring ongoing community services to facilitate their return to everyday activities [2-4]. The majority of research to date is predominantly focused on male participants likely due to males having a higher incidence of TBI than women. Information regarding the effect of TBI on women's health is limited. Further, it is known that women with disability lack sufficient medical care compared to women without a disability [5]. The examination of health outcomes based on gender may reflect important differences post TBI and help inform health service decision-makers.

There is a growing body of literature examining gender differences in outcome post TBI, however, findings are inconsistent. In a meta-analysis by Farace and Alves [6] women reported worse outcomes than men in 85% of measured variables; this was further supported in a recent systematic review [7] but with emphasis on worse outcomes in studies using older women. Conversely, others report superior results for community integration for women [8,9]. Further, studies investigating gender and its influence on functional outcome, found no difference between men and women [10-12]. Though similarities in functional outcome are apparent, consistent differences in TBI symptoms between men and women have been identified.

Literature examining gender differences in neuropsychological symptoms post TBI is more extensive than literature examining community integration and functional recovery. Numerous studies of gender differences in cognitive and psychological test performance have illustrated important distinctions. Executive function has been found to be superior in women compared to men post TBI [13-15] as well as memory and cognitive flexibility [16-18]. Whiteneck et al [19] studied outcomes after hospitalization for TBI one-year post-injury. Women were more likely to report symptoms such as fatigue, headaches, and balance problems compared to men. Headaches have been reported more frequently by women than men post TBI in several studies [20-22]. Hibbard et al [21] examined the prevalence of health issues 10 years post TBI and found being female and increased age were significant predictors for thyroid conditions, sleep difficulties, loss of urinary control and arthritis. Further, gender differences in symptoms of depression and anxiety have been reported with women demonstrating consistently worse outcomes [18,20,23]. These results highlight continued difficulty with cognitive and emotional functioning long after injury and reflect the need for ongoing health service programs.

This study is part of a larger study which was designed to examine long term outcomes post TBI [24]. A study objective was to examine the impact of factors such as gender and injury severity on long term outcomes. This study examines gender differences on self-reported symptoms 7 to 24 years after a moderate to severe traumatic brain injury and examines gender differences in the degree to which reported symptoms present as a problem for daily functioning.

## Methods

### Participants

Participants were from a retrospective cohort study on long term function post TBI [24]. Participants (n = 306) had sustained a moderate to severe TBI and were discharged from a rehabilitation treatment centre in Pittsburgh, Pennsylvania. The criteria for inclusion were: (1) participants sustained the TBI at age 14 or older; (2) participants had specific head injury ICD-8 and ICD-9 codes (800-801.9, 803-804.9, 850-854.9: concussion; cerebral laceration or contusion; subarachnoid, subdural, or extra-dural hemorrhage or injury; and fractures to the skull); (3) participants lived within a 150 mile radius from the HealthSouth Harmarville Rehabilitation Hospital in Pennsylvania. Ethics approval was granted from the research ethics boards at the University of Toronto and the Healthsouth Rehabilitation Hospital.

### Data collection procedure

The data collection procedure is described in detail in the original study. Essentially 642 individuals identified through medical record review were eligible for an interview, but 42 were not traceable, 128 were deceased, and 82 had moved. The remaining 390 eligible individuals were sent a letter introducing the study and requesting their participation. If the individual did not wish to be contacted, they were to call the project office's confidential phone line. Seven days later a trained interviewer phoned these individuals to arrange for a home interview, at which time informed consent was obtained. Among the 390 persons eligible for an interview, 30 were lost to follow up and 52 refused. Interviews were scheduled for 308 individuals. Physical, cognitive or communication limitations prevented 22 individuals from participating in the interview. In 20 of these cases, the individual nominated a caregiver/friend/family member to complete the interview. The data used in this study was collected from interviews with 286 survivors and 20 informants.

### Assessments

Information relating to demographics (e.g. age, martial status, and level of education) and injury characteristics (e.g. time since, cause, and severity) was taken from medical records and the History and Physical Medicine and Rehabilitation Evaluation, conducted by the admitting physicians in the rehabilitation hospital. In older records the Glasgow Coma Scale was inconsistently completed. To be consistent, information about length of unconsciousness or post-traumatic amnesia (PTA) was used to establish injury severity. Estimates of the durations of coma and PTA were derived from the admitting physician's account of the acute care phase.

The instrument used to collect the data was the Problem Checklist (PCL) from the Head Injury Family Interview (HIFI) [25]. The PCL is a two-part, self-report measure of 43 symptoms that are commonly experienced after a brain injury. In part A, participants answer 'yes' or 'no' to whether they currently experience one of the symptoms such as headaches, irritability, or forgetfulness. If the participant endorses the symptom in part A, they are then asked to rate 'how much of a problem this symptom presents in their daily functioning' (part B). Rating is done using a seven point Likert scale with one indicating 'no problem' to seven indicating 'severe problem'. Good internal validity (Cronbach's ά = 0.91 to 0.78) and construct validity (r = 0.46 to 0.37) were found [25]. Because the PCL is self-report, Kay and colleagues [25] compared reports from individuals with TBI to those obtained from family members in order to validate responses; they found significant correlations (p < 0.001) for all scales. Paniak et al [26] found the PCL was sensitive to differences in symptom reporting between individuals with TBI and those without (p < 0.001).

### Analyses

Descriptive statistics were generated, such as frequency distributions, measures of central tendencies and dispersion by gender. Group differences defined by gender were produced using Chi-square for dichotomous variables and Wilcoxon two-tailed test for continuous data from Part B of the PCL. We completed analysis for multiple comparisons using Bonferonni's adjustment with correction for correlation between observations [27,28]. Significance for adjusted differences was set at p ≤ 0.025. Calculations were performed using SAS statistical software package.

## Results

### Sociodemographic information

Of the 306 participants, there were 213 men and 93 women. No significant differences were found between men and women for demographic and injury-related characteristics, which are displayed in Table 1. The mean age at injury was 29 for men and 32 for women and the mean age at follow-up was 43 years for men and 46 years for women. Men and women both had a mean of 14 years from time of injury to follow-up, with the range being 8-24 years for men and 7-24 years for women. Both men and women had a mean of 12 years of education prior to injury. Data on loss of consciousness (LOC) were available for most participants (182 men and 76 women). LOC for greater than 24 hours was noted for 76% of men and 70% of women. LOC for greater than one week was found in 49% of men and 47% of women. Individuals with a LOC of less than one hour included 13% of men and 17% of women.

**Table 1:** Demographic and injury characteristics (Men = 213, Women = 93)

Characteristics	N		N	
	
	M(SD)	W(SD)	M(%)	W(%)
Age at injury	29(12)	32(15)		

Current age	43(12)	46(15)		

Years since injury	14(4.3)	14(4.6)		

Year of education prior to injury	12(1.9)	12(2.5)		

Marital Status at injury				
• Married			73(34)	34(37)
• Not married			124(58)	45(49)
• Separated/divorced/widowed			15(7)	12(13)

Loss of consciousness				
• Less than 1 hour			24(13)	13(17)
• Greater than 1 day			138(76)	53(70)
• Greater than 1 week			89(49)	36(47)

### Gender differences on symptoms reported - Part A of the PCL

Women reported being forgetful (70.1%), difficulty remembering the right word (63.6%), poor balance (62.5%), visual problems (57.9%) and irritability (57.4%) most frequently. Similar findings were found for men with forgetfulness (72.6%), irritability (66.1%), poor balance (65%), doing things slowly (62.5%) and difficulty remembering the right word (58.7%) reported most frequently. A summary of gender differences on self reported symptoms in part A of the PCL can be found in Table 2. Significantly more men than women reported difficulty setting realistic goals (p < 0.02), high sex drive and restlessness (p < 0.01), whereas significantly more women than men reported loss of confidence (p < 0.02) as a problem.

**Table 2:** Chi-Square Gender Differences on the Problem Checklist for Symptoms Reported With Over 30% Frequency - Part A of Checklist

Symptom	Male - % Yes	Female - % Yes	P-Value*
1a Visual	51.2	57.9	0.29

3a Balance	65.0	62.5	0.67

4a Slow	62.5	53.4	0.14

5a Words	43.3	34.4	0.15

6a Coordination	56.1	50.5	0.38

7a Tired	44.8	49.4	0.47

8a Headaches	29.6	42.5	0.03

9a Dizziness	19.1	30.5	0.03

13a Word finding	58.7	63.6	0.43

14a Wordy	38.8	44.3	0.37

15a Distracted	44.7	39.7	0.42

16a Concentration	47.2	40.9	0.31

17a Forgetful	72.6	70.1	0.66

18a Think clearly	37.3	37.6	0.95

19a Planning	30.5	33.3	0.63

**20a Set Goals**	**37.3**	**23.2**	**0.02**

21a Finishing	33.3	32.1	0.84

22a Apathy	42.0	38.6	0.58

23a Initiative	38.6	30.6	0.19

24a Irritability	66.1	57.4	0.15

**25a Restlessness**	**52.9**	**37.0**	**0.01**

26a Temper	51.4	52.8	0.83

27a Mood Swings	37.7	44.9	0.24

31a Bored	45.7	38.3	0.24

32a Complain	33.6	36.0	0.69

33a Dependent	46.6	40.2	0.31

35a Anxiety	48.0	52.8	0.44

36a Depression	47.2	48.8	0.80

37a Lonely	38.3	30.2	0.19

**38a Confidence**	**37.5**	**50.0**	**0.02**

**42a High sex drive**	**21.39**	**09.52**	**0.01**

43a Personality	32.1	40.0	0.20

*adjusted p value = 0.02

### Gender differences in problematic symptoms - Part B of the PCL

A summary of gender differences on the mean scores from part B of the PCL can be found in Table 3. For men, the five most frequently reported problems were loss of confidence, sleep difficulty, difficulty thinking clearly, needing supervision, and fatigue. For women, needing supervision, lack of initiative, difficulty planning and organizing things, difficulty thinking clearly, and difficulty setting realistic goals were the most frequently reported problems. Men reported sensitivity to noise (p < 0.02) and sleep disturbances (p < 0.02) as having a greater impact on daily functioning compared to women. Symptoms presenting significantly more of a problem in daily functioning for women compared to men was lack of initiative (p < 0.02) and needing supervision (p < 0.02).

**Table 3:** Wilcoxon Gender Differences on the Problem Checklist for Symptom Impact on Daily Function - Part B of Checklist

Symptom	Mean: Male	Mean: Female	p-value*
**2b Hearing**	**3.3**	**2.4**	**0.01**

3b Balance	3.8	3.7	0.64

4b Slow	3.5	3.8	0.44

5b Words	3.6	3.3	0.43

6b Coordination	4.0	4.0	0.85

7b Tired	4.0	3.5	0.21

8b Headaches	3.4	3.1	0.48

9b Dizziness	3.4	3.1	0.58

**10b Noise**	**3.5**	**2.6**	**0.02**

11b Light	3.8	3.1	0.23

13b Word finding	3.6	3.44	0.37

14b Wordy	3.9	3.6	0.38

15b Distracted	3.5	4.0	0.19

16b Concentration	4.0	3.8	0.69

17b Forgetful	3.9	3.7	0.44

18b Think clearly	4.1	4.1	0.93

19b Planning	3.9	4.2	0.45

20b Set Goals	3.8	4.1	0.54

21b Finishing	3.9	4.1	0.78

22b Apathy	3.4	4.0	0.11

**23b Initiative**	**3.7**	**4.5**	**0.02**

24b Irritability	3.3	3.3	0.87

25b Restlessness	3.6	3.5	0.85

26b Temper	3.4	3.6	0.47

27b Mood Swings	3.9	3.5	0.35

28b Emotional	3.6	3.7	0.78

30b Violent	3.5	3.4	1.00

31b Bored	3.8	3.7	0.74

32b Complain	3.4	3.2	0.68

33b Dependent	3.7	4.0	0.51

**34b Supervision**	**4.0**	**5.5**	**0.02**

35b Anxiety	3.1	3.4	0.24

36b Depression	3.6	3.5	0.93

37b Lonely	3.7	3.8	0.70

38b Confidence	4.3	3.9	0.33

**40b Sleep**	**4.2**	**3.1**	**0.02**

41b Low sex drive	3.7	3.1	0.37

**42b High sex drive**	**2.2**	**3.5**	**0.01**

43b Personality	3.1	3.2	0.78

*adjusted p value = 0.025

## Discussion

This is the only study to our knowledge that has investigated gender differences on self reported symptoms and symptom impact on daily living many years post injury. We found several similarities on self reported symptoms between men and women but also significant differences. These differences can provide information to health care providers for the planning and delivery of care for individuals with a TBI.

Four of the five most reported symptoms were the same for men and women, highlighting similarities in symptoms experienced after TBI. These symptoms include being forgetful, irritability, poor balance, and word finding difficulties. This finding is consistent with previous studies of symptom prevalence [19,22,29]. Poor balance was the third most reported symptom for men and women after TBI which has safety implications, particularly since the main cause of brain injury in older adults is falls [2]. The diversity of self-reported symptoms reported in the chronic stage of recovery indicates the need for ongoing services to provide programming which includes cognitive, physical, psychosocial components in order to facilitate successful integration into community life.

Significantly more women reported headaches and dizziness than men. This difference is supported by previous studies with TBI survivors [19,21,22] as well as in the general population [30,31]. Therefore, the distinction found in our study may not be directly related to the injury, but rather reflect what is found in the general population. Why women comprise a larger proportion of individuals reporting these symptoms is not clear, although there is some evidence of neurophysiological factors [21,32].

Additionally, headaches can be attributed to soft tissue injury of the neck and upper body; women may be more susceptible to trauma of soft tissue during acceleration-deceleration injuries due to higher head to body mass ratio compared to men [33]. Regardless, headaches and dizziness are associated with difficulty performing daily activities [19,34,35] and vocational tasks [36,37]. As such, these symptoms should be a priority for treatment intervention, pharmacological and educational, along the continuum of care.

Men reported hearing/noise difficulties and sleep disturbances as significantly more problematic than women. Several studies have found sensitivity to noise as a prominent sequelae of TBI [20,22,38] and a factor in poor functional outcome [19,20,39]. The meta-analysis by Farace and Alves [6] found significantly more men than women reported hearing related problems. Noise sensitivity may impact social and vocational involvement and success, known areas of difficulty post TBI [3,19,40]. Clinicians may need to incorporate environmental assessments into discharge and return to work planning to detect possible noise/hearing irritants that could impact effective community integration.

Sleep disturbances are prevalent in both men and women post TBI [41-43] and can complicate recovery [44,45]. Sleep disturbances are also associated with depression, anxiety, and poor outcome on cognitive measures [44,46,47]. Self-report of sleep disturbances makes it difficult to determine whether the issue relates to the TBI itself or to secondary complications such as depression, stress or pain [48-50]. In our study men reported sleep disturbances as significantly more problematic for daily living compared to women. One explanation may be the effect of sleep deprivation on paid work as more men in our study were working outside the home than women. The literature is inconclusive regarding gender differences and subsequent causes of sleep disturbances [41,44]. Vigilance to the pervasive impact of sleep disturbances for individuals with TBI is imperative. The complex interrelationship between TBI, cognitive and psychological symptoms and sleep disturbances further supports the need for comprehensive assessment and treatment programs.

Self-report measures such as the one used in this study can capture the socio-medical perspective of health such as social influence on illness and health reporting behaviours, which may account for some of our results [51]. The fact that significantly more men reported high sex drive than women may be due to social acceptance and willingness to report rather than an organic brain disturbance caused by the injury. In addition, women reported needing supervision as significantly more problematic than men. Again, this may reflect the social pressure of sustaining care-taker and home-maker roles without feeling able to ask for assistance. Additionally, environmental constraints may affect a woman's ability to perform and balance home and community activities, thus the need for assistance [5].

## Limitations

A main limitation of this study is the lack of a control group to ensure symptoms reported are due to the TBI and not a reflection of differences seen in the general population. In addition, due to the many comparisons in our study, it is possible that some of our significant differences are spurious; however we did use an adjusted p value to minimize type 1 error. Further, one participant's interpretation of a symptom, such as 'thinking clearly' may be different from another's, which could influence the frequency and magnitude of symptom reporting. Self-report measures may not reflect true symptomology as measured by standardized neuro-cognitive tests; however, our goal was to document reported symptoms and their effect on daily functioning rather than measure cognitive deficits. Our participants were a minimum of seven years post injury, however the PCL was developed using a sample of individuals three years post injury; there may be differences in symptoms and symptom impact when measured at later stages of chronicity. Since some of our participants were classified with milder injury, findings may not accurately represent this sub-population.

## Conclusion

In conclusion, men and women experience symptoms that are problematic many years after a TBI. We found similarities and differences in reported symptoms based on gender. Future research examining gender differences in how cognitive and physical symptoms influence community integration and performance in day to day activities would contribute to the understanding of the dynamic interaction between these concepts. Since some of our participants were classified with milder injury, findings may not accurately represent this sub-population.

## Competing interests

The authors declare that they have no competing interests.

## Authors' contributions

AC was responsible for the concept, design, coordination of the study as well as data analysis/interpretation and manuscript revisions.

JEH was responsible for data analysis and interpretation as well as the writing and revisions of this manuscript.

GR was responsible for the concept, design, and coordination of the study and manuscript approval.

SC was responsible for the concept, design, and coordination of the study as well as manuscript approval.

KE was responsible for preliminary data analysis and interpretation and manuscript approval.

## Pre-publication history

The pre-publication history for this paper can be accessed here:

http://www.biomedcentral.com/1471-2377/10/102/prepub
